# Supplementary material for: Germ-Free Conditions Modulate Host Purine Metabolism, Exacerbating Adenine-Induced Kidney Damage
Source: Toxins (Basel). 2020 Aug 26;12(9):547. doi: 10.3390/toxins12090547 (PMC7551802; doi:10.3390/toxins12090547)
Supplement: Supplementary file 1 [file toxins-12-00547-s001.pdf]

Supplementary Materials: Germ-Free Conditions Modulate Host Purine Metabolism, Exacerbating Adenine-Induced Kidney Damage

Eikan Mishima, Mariko Ichijo, Takeshi Kawabe, Koichi Kikuchi, Yukako Akiyama, Takafumi Toyohara, Takehiro Suzuki, Chitose Suzuki, Atsuko Asao, Naoto Ishii, Shinji Fukuda and Takaaki Abe

Table S1. Taqman probes used for quantitative PCR.

Gene	Product Number
Mouse Xdh	Mm00442110_m1
Mouse Aprt	Mm00782508_s1
Mouse Hprt	Mm03024075_m1
Mouse Prps1	Mm00727494_s1
Mouse Pnp1	Mm00840006_m1
Mouse Tgfb1	Mm01178820_m1
Mouse Col1a1	Mm00801666_g1
Mouse Fn1	Mm01256744_m1
Mouse Il17a	Mm00439618_m1
Mouse Il17f	Mm00521423_m1
Mouse Il23a	Mm00518984_m1
Mouse Il1a	Mm00439620_m1
Mouse Emr1 (Adgre1)	Mm00802529_m1
Mouse Tnf	Mm00443258_m1
18S rRNA	Hs99999901_s1

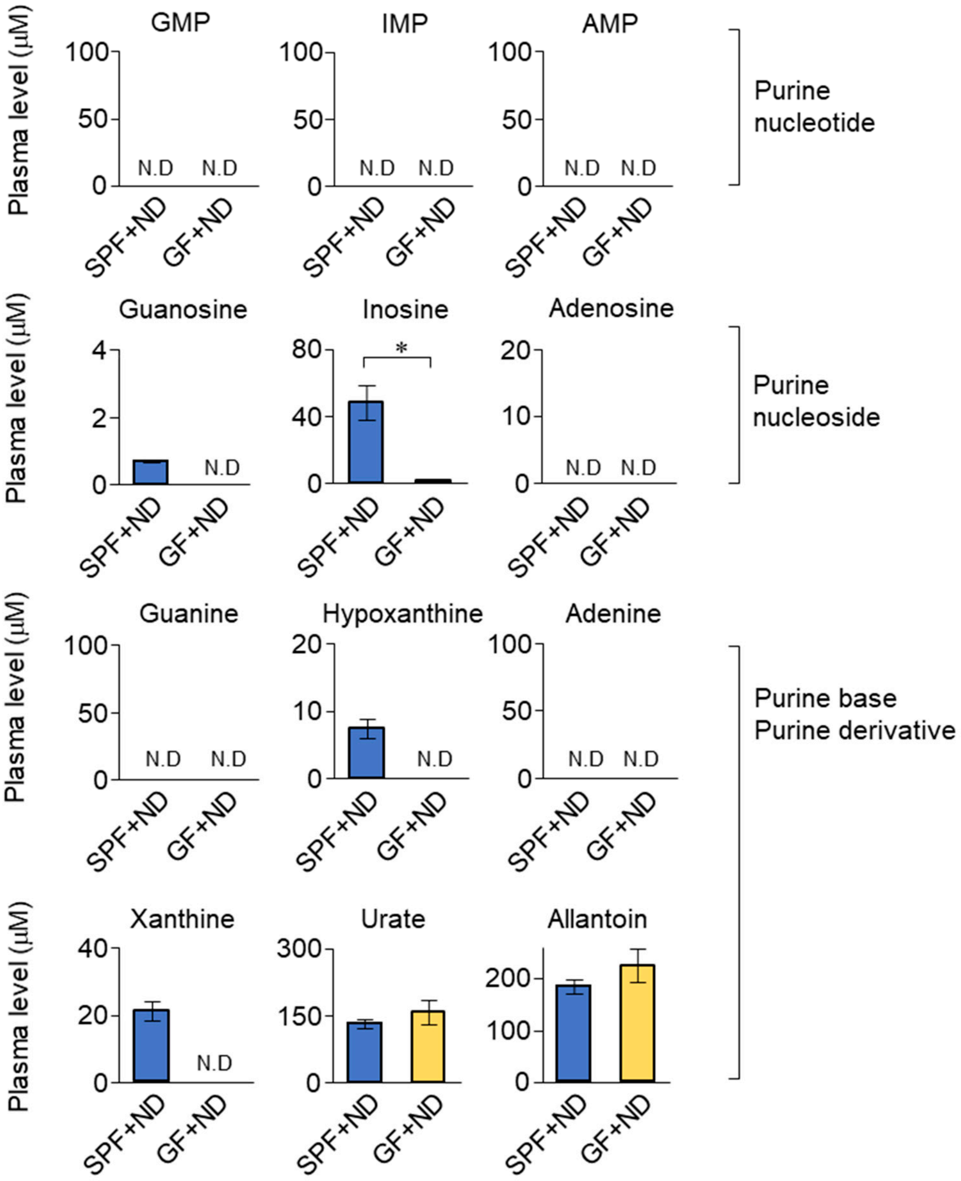

Figure S1. Plasma nucleotide metabolites in GF+ND and SPF+ND mice. * $p < 0.05$ compared between indicated groups (*t*-test). N.D, not detectable.

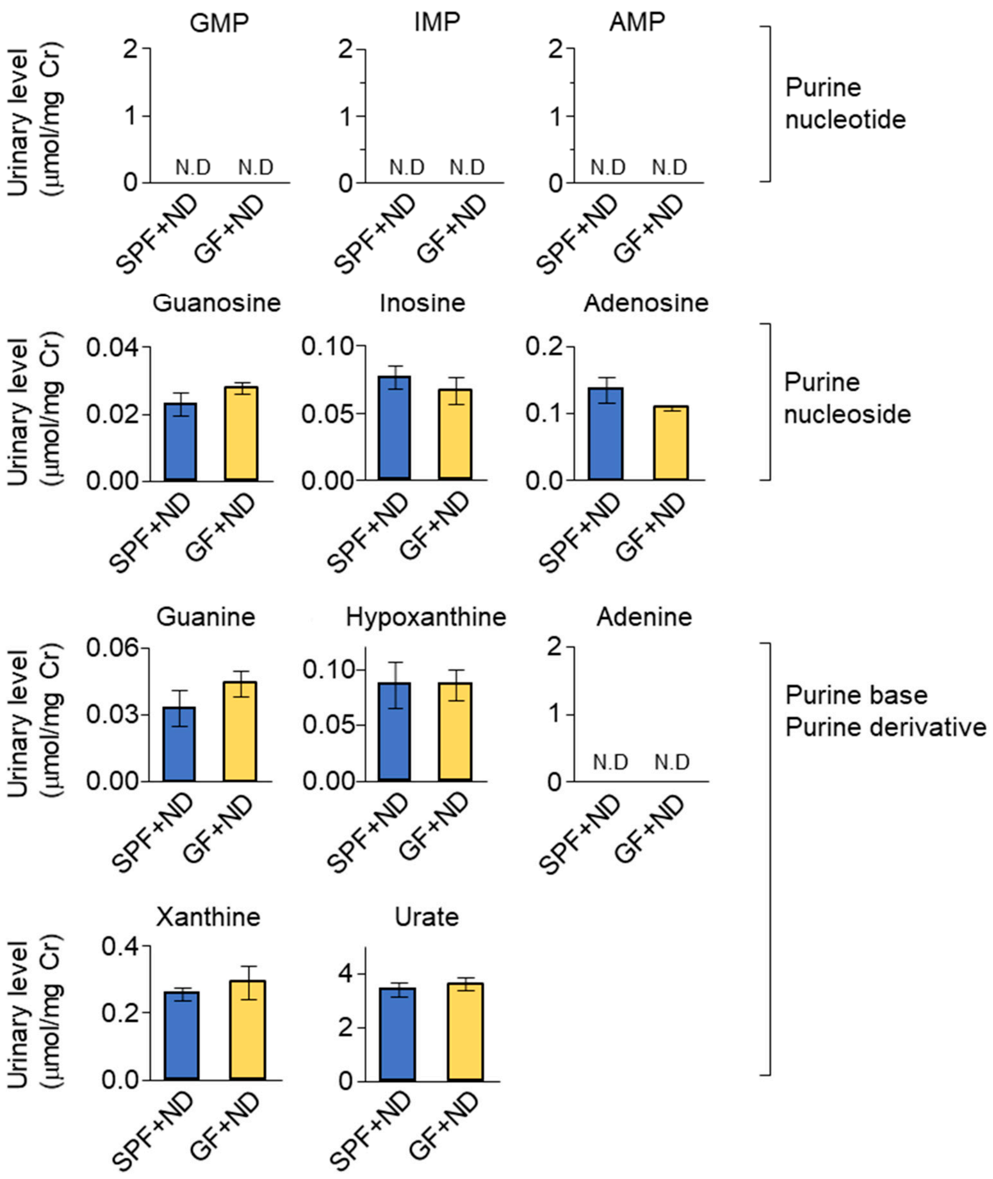

Figure S2. Urinary nucleotide metabolites in GF+ND and SPF+ND mice. Urinary concentrations were corrected urinary creatinine (μmol/mg urinary creatinine). * $p < 0.05$ compared between indicated groups (*t*-test). N.D, not detectable.
